# A guide to selecting high-performing antibodies for RNA-binding protein TIA1 for use in Western Blot, immunoprecipitation and immunofluorescence

**DOI:** 10.12688/f1000research.133645.2

**Published:** 2024-04-08

**Authors:** Maryam Fotouhi, Donovan Worrall, Riham Ayoubi, Kathleen Southern, Peter S. McPherson, Carl Laflamme, NeuroSGC/YCharOS/EDDU collaborative group, ABIF Consortium

**Affiliations:** 1 Department of Neurology and Neurosurgery, Structural Genomics Consortium, The Montreal Neurological Institute, McGill University, Montreal, Québec, H3A 2B4, Canada

**Keywords:** Uniprot ID P31483, TIA1, RNA-binding protein TIA1, antibody characterization, antibody validation, Western Blot, immunoprecipitation, immunofluorescence

## Abstract

A member of the RNA-binding protein family, T-cell intracellular antigen-1 (TIA1) regulates mRNA translation and splicing as well as cellular stress by promoting stress granule formation. Variants of the *TIA1* gene have implications in neurogenerative disorders including amyotrophic lateral sclerosis (ALS) and frontotemporal dementia (FTD). Reproducible research on TIA1 would be enhanced with the availability of high-quality anti-TIA1 antibodies. In this study, we characterized twelve TIA1 commercial antibodies for Western Blot, immunoprecipitation, and immunofluorescence using a standardized experimental protocol based on comparing read-outs in knockout cell lines and isogenic parental controls. We identified many high-performing antibodies and encourage readers to use this report as a guide to select the most appropriate antibody for their specific needs.

## Introduction

T-cell intracellular antigen 1, or TIA1, is a cytotoxic granule-associated RNA-binding protein involved in regulating alternative pre-mRNA splicing and mRNA translation when bound to 3’ uridine-rich RNA sequences. ^1^^–^^4^ Suppressing translation in environmentally stressed cells and promoting stress granule formation, TIA1 modulates cellular response to stress and inflammation. ^5^^,^^6^

Comparable to other RNA-binding proteins, disrupting the function of TIA1 can lead to various diseases including cancer, ^7^^–^^9^ autoimmune diseases ^10^ and neurodegenerative disorders. ^11^^–^^14^ Studies have demonstrated that mutations to the *TIA1* gene may delay the disassembly of stress granule, resulting in insoluble and immobile stress granules, a clinical feature of ALS and FTD. ^6^^,^^11^ Significant efforts are required to further elucidate the relationship between dysregulated RNA metabolism and ALS/FTD pathogenesis which may lead to novel therapeutic discoveries. ^11^^,^^15^

Mechanistic studies would be greatly facilitated with the availability of high-quality antibodies. Here, we compared the performance of a range of commercially-available antibodies for TIA1 and identified high-performing antibodies for Western Blot, immunoprecipitation and immunofluorescence, enabling biochemical and cellular assessment of TIA1 properties and function.

## Results and discussion

Our standard protocol involves comparing readouts from wild-type (WT) and knockout (KO) cells. ^16^^–^^23^ The first step was to identify a cell line(s) that expresses sufficient levels of TIA1 to generate a measurable signal. To this end, we examined the DepMap transcriptomics database to identify all cell lines that express the target at levels greater than 2.5 log _2_ (transcripts per million “TPM” + 1), which we have found to be a suitable cut-off (Cancer Dependency Map Portal, RRID:SCR_017655). Commercially available HAP1 cells expressed the *TIA1* transcript at RNA levels above the average range of cancer cells analyzed. Parental and *TIA1* KO HAP1 cells were obtained from Horizon Discovery ( Table 1).

**Table 1.** Summary of the cell lines used.

Institution	Catalog number	RRID (Cellosaurus)	Cell line	Genotype
Horizon Discovery	C631	CVCL_Y019	HAP1	WT
Horizon Discovery	HZGHC003048C010	CVCL_TS30	HAP1	*TIA1* KO

**Figure 1.**
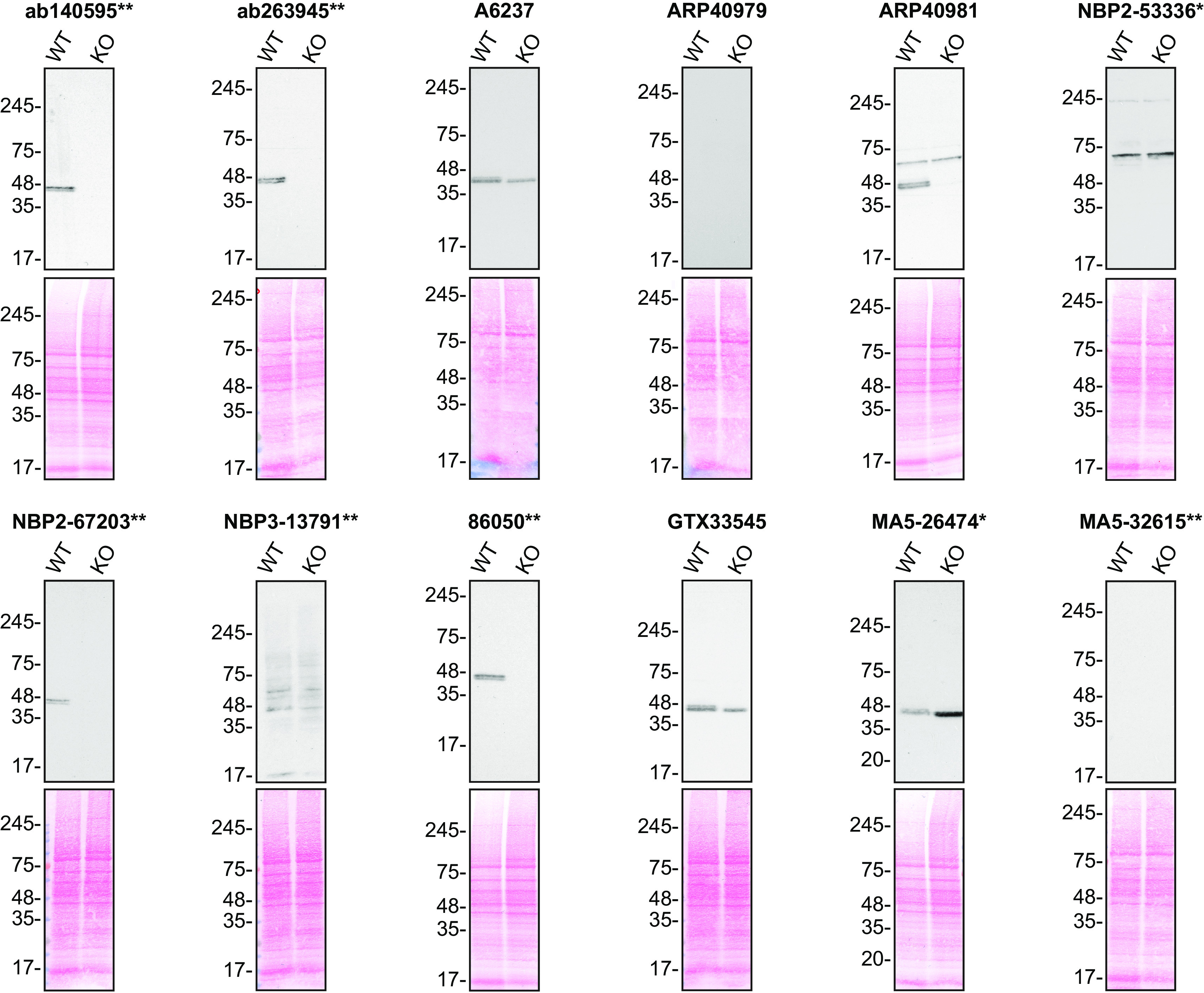
TIA1 antibody screening by Western Blot. Lysates of HAP1 (WT and *TIA1* KO) were prepared and 50 μg of protein were processed for Western Blot with the indicated TIA1 antibodies. The Ponceau stained transfers of each blot are presented to show equal loading of WT and KO lysates and protein transfer efficiency from the acrylamide gels to the nitrocellulose membrane. Antibody dilutions were chosen according to the recommendations of the antibody supplier. Antibody dilution used: ab140595** at 1/5000; ab263945** at 1/1000; A6237 at 1/1000; ARP40979 at 1/500; ARP40981 at 1/200; NBP2-53336* at 1/1000; NBP2-67203** at 1/1000; NBP3-13791** at 1/500; 86050** at 1/1000; GTX33545 at 1/500; MA5-26474* at 1/2000; and MA5-32615** at 1/500. Predicted band size: 43 kDa. *=monoclonal antibody, **=recombinant antibody.

For Western Blot experiments, we resolved proteins from WT and *TIA1* KO cell extracts and probed them side-by-side with all antibodies in parallel ( Figure 1). ^17^^–^^23^

**Figure 2.**
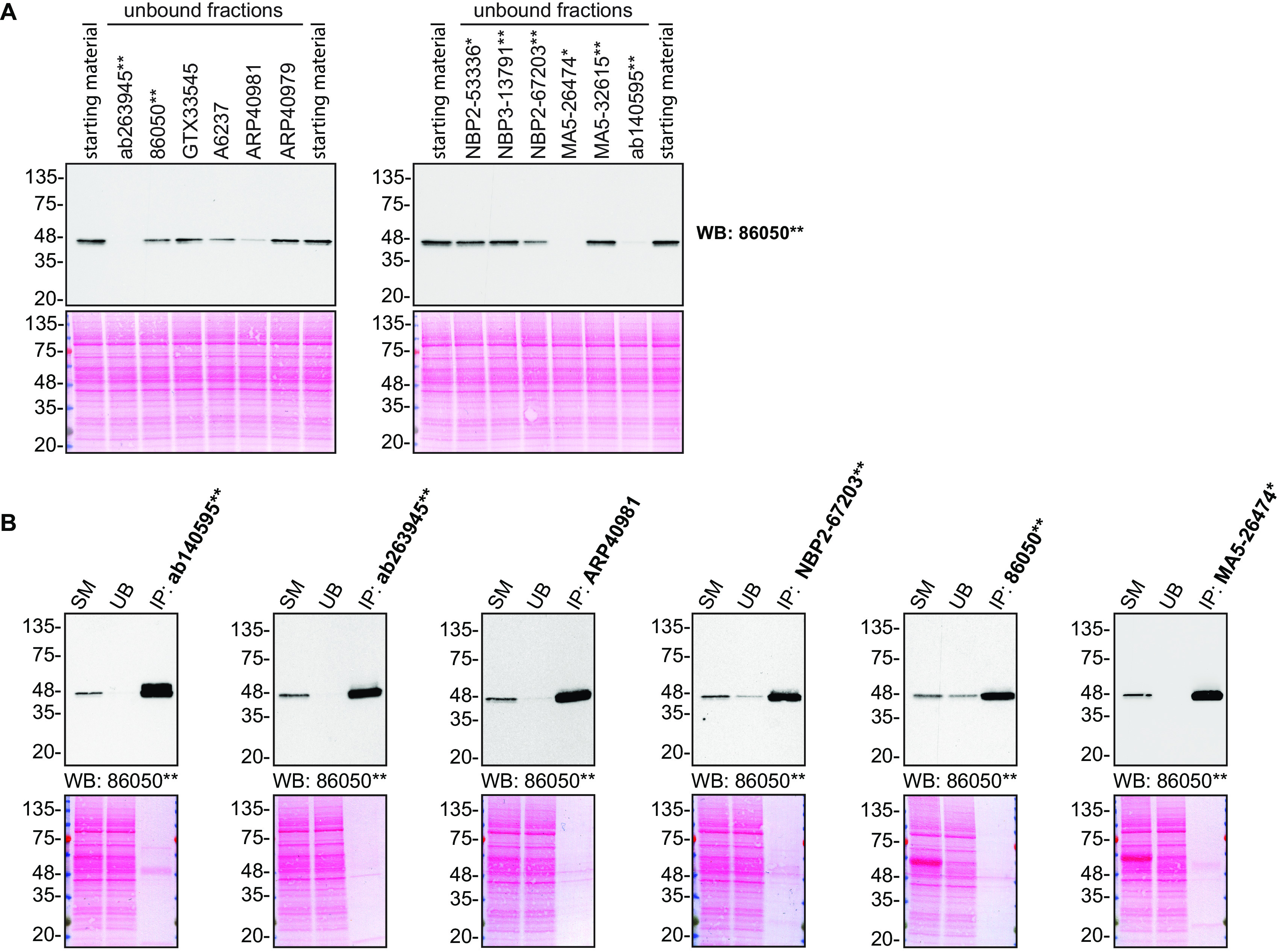
TIA1 antibody screening by immunoprecipitation. HAP1 lysates were prepared, and IP was performed using 1.0 μg of the indicated TIA1 antibodies pre-coupled to Dynabeads protein G or protein A. (A) Ability of the antibodies to capture TIA1 was assessed by comparing the level of protein available in the starting material to the level remaining in the unbound fraction. (B) The immunoprecipitates for antibodies which could immunocapture TIA1 in (A) are shown. For Western Blot, 86050** was used at 1/1000 in A) and B). The Ponceau stained transfers of each blot are shown. SM=4% starting material; UB=4% unbound fraction; IP=immunoprecipitate. *=monoclonal antibody, **=recombinant antibody.

For immunoprecipitation experiments, we used the antibodies to immunopurify TIA1 from HAP1 cell extracts. The performance of each antibody was evaluated by detecting the TIA1 protein in extracts, in the immunodepleted extracts and in the immunoprecipitates ( Figure 2). ^17^^–^^23^

**Figure 3.**
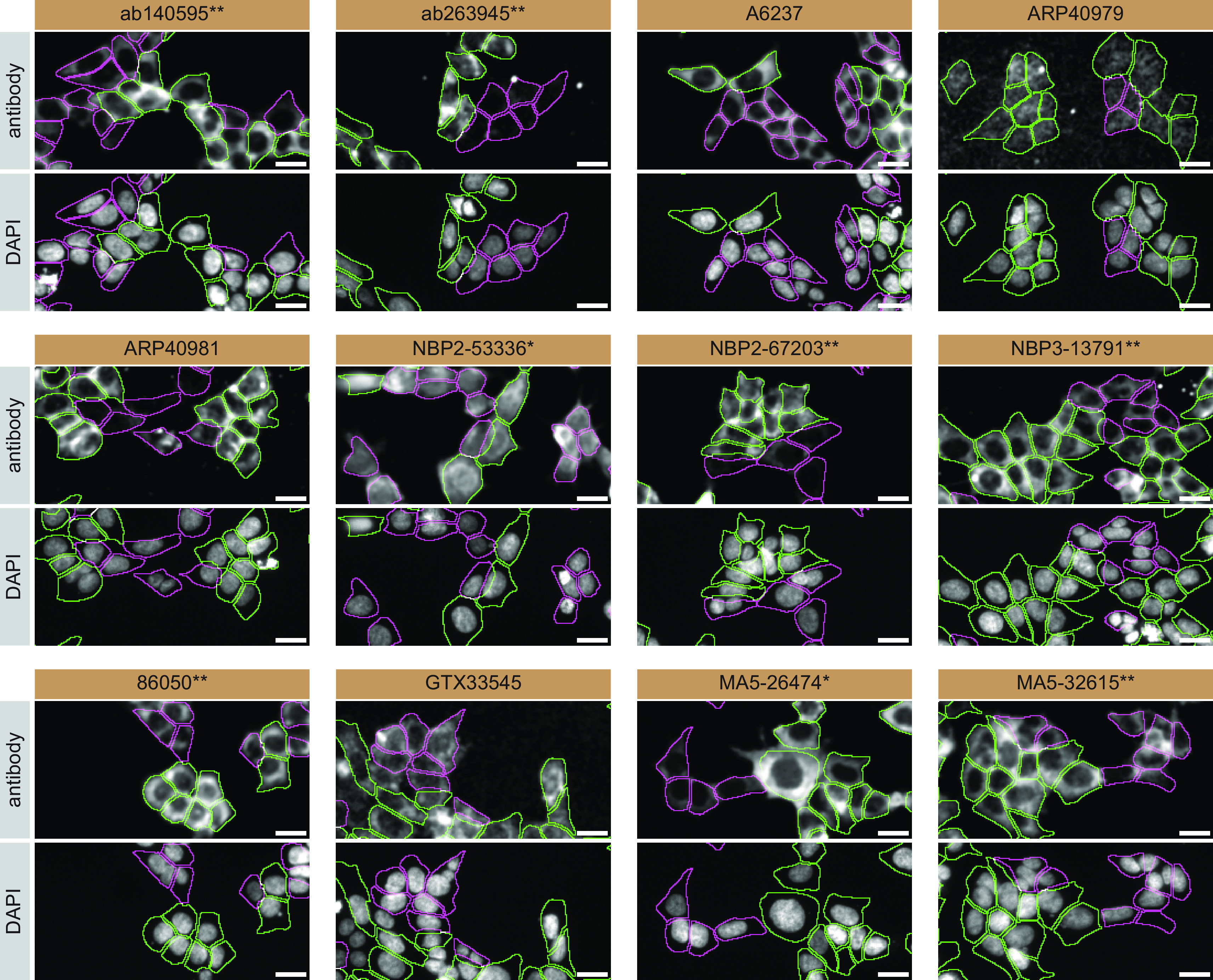
TIA1 antibody screening by immunofluorescence. HAP1 WT and TIA1 KO cells were labelled with a green or a far-red fluorescent dye, respectively. WT and KO cells were mixed and plated to a 1:1 ratio in a 96-well plate with optically clear flat-bottom. Cells were stained with the indicated TIA1 antibodies and with the corresponding Alexa-fluor 555 coupled secondary antibody including DAPI. Acquisition of the blue (nucleus-DAPI), green (identification of WT cells), red (antibody staining) and far-red (identification of KO cells) channels was performed. Representative images of the merged blue and red (grayscale) channels are shown. WT and KO cells are outlined with green and magenta dashed line, respectively. Antibody dilutions were chosen according to the recommendations of the antibody supplier. Exceptions were given to antibodies ab140595**, A6237, NBP2-67203**, and GTX33545, which were titrated to their respective concentrations found bellow, as the signals were too weak when following the suppliers' recommendations. When the concentrations were not indicated by the supplier, which was the case for antibodies ab263945**, ARP40979, ARP40981, and NBP3-13791** we tested antibodies at 1/300, 1/500, 1/500 and 1/200, respectively. At these concentrations, the signal from each antibody was in the range of detection of the microscope used. Antibody dilution used: ab140595** at 1/600; ab263945** at 1/300; A6237 at 1/1000; ARP40979 at 1/500; ARP40981 at 1/500; NBP2-53336* at 1/100; NBP2-67203** at 1/1000; NBP3-13791** at 1/200; 86050** at 1/100; GTX33545 at 1/800; MA5-26474* at 1/1000; and MA5-32615** at 1/100. Bars = 10 μm. *=monoclonal antibody, **=recombinant antibody.

For immunofluorescence, as described previously, antibodies were screened using a mosaic strategy. ^24^ In brief, we plated WT and KO cells together in the same well and imaged both cell types in the same field of view to reduce staining, imaging and image analysis bias ( Figure 3).

In conclusion, we have screened TIA1 commercial antibodies by Western Blot, immunoprecipitation and immunofluorescence. Several high-quality antibodies that successfully detect TIA1 under our standardized experimental conditions can be identified. In our effort to address the antibody reliability and reproducibility challenges in scientific research, the authors recommend the antibodies that demonstrated to be underperforming under our standard procedure be removed from the commercial antibody market. However, the authors do not engage in result analysis or offer explicit antibody recommendations. A limitation of this study is the use of universal protocols - any conclusions remain relevant within the confines of the experimental setup and cell line used in this study. Our primary aim is to deliver top-tier data to the scientific community, grounded in Open Science principles. This empowers experts to interpret the characterization data independently, enabling them to make informed choices regarding the most suitable antibodies for their specific experimental needs. Guidelines on how to interpret antibody characterization data found in this study are featured on the YCharOS gateway. ^25^ The underlying data can be found on Zenodo open access repository. ^26^^,^^27^

## Methods

### Antibodies

All tested TIA1 antibodies are listed in Table 2, together with their corresponding Research Resource Identifiers, or RRID, to ensure the antibodies are cited properly. ^28^ Peroxidase-conjugated goat anti-rabbit and anti-mouse antibodies are from Thermo Fisher Scientific (cat. number 65-6120 and 62-6520). Alexa-555-conjugated goat anti-rabbit and anti-mouse secondary antibodies are from Thermo Fisher Scientific (cat. number A21429 and A21424).

**Table 2.** Summary of the TIA1 antibodies tested.

Company	Catalog number	Lot number	RRID (Antibody Registry)	Clonality	Clone ID	Host	Concentration (μg/μl)	Vendors recommended applications
Abcam	ab140595 **	GR223312-17	AB_2687963	recombinant-mono	EPR9304	rabbit	0.665	Wb, IP, IF
Abcam	ab263945 **	GR3297000-3	AB_2885132	recombinant-mono	EPR22999-80	rabbit	0.599	Wb, IP
Abclonal	A6237	18980101	AB_2766845	polyclonal	-	rabbit	2.920	Wb, IF
Aviva Systems Biology	ARP40979	QC10247-90408	AB_2048416	polyclonal	-	rabbit	0.500	Wb
Aviva Systems Biology	ARP40981	QC10249-41152	AB_938457	polyclonal	-	rabbit	0.500	Wb, IP
Bio-Techne	NBP2-53336 *	7072-1p210821	AB_2885158	monoclonal	TIA1/1313	mouse	0.200	Wb, IF
Bio-Techne	NBP2-67203 **	H00823	AB_2927744 ^1^	recombinant-mono	JM42-11	rabbit	1.000	Wb, IP, IF
Bio-Techne	NBP3-13791 **	7072-2P210904	AB_2927743 ^1^	recombinant-mono	TIA1/1352R	rabbit	0.200	other
Cell Signaling Technology	86050 **	1	AB_2800070	recombinant-mono	D1Q3K	rabbit	0.002	Wb, IP
GeneTex	GTX33545	822104511	AB_2887719	polyclonal	-	rabbit	0.820	Wb, IF
Thermo Fisher Scientific	MA5-26474 *	VL3152368	AB_2725518	monoclonal	OTI1D7	mouse	1.000	Wb, IF
Thermo Fisher Scientific	MA5-32615 **	VL3152613	AB_2809892	recombinant-mono	JM42-11	rabbit	1.000	Wb, IP, IF

Wb=Western blot; IF= immunofluorescence; IP=immunoprecipitation.

* = monoclonal antibody.

** = recombinant antibody.

^1^ refers to RRID recently added to the Antibody Registry (in February 2023), they will be available on their website in the coming weeks.

### Cell culture

Both HAP1 WT and *TIA1* KO cell lines used are listed in Table 1, together with their corresponding RRID, to ensure the cell lines are cited properly. ^29^ Cells were cultured in DMEM high-glucose (GE Healthcare cat. number SH30081.01) containing 10% fetal bovine serum (Wisent, cat. number 080450), 2 mM L-glutamine (Wisent cat. number 609065), 100 IU penicillin and 100 μg/mL streptomycin (Wisent cat. number 450201).

### Antibody screening by Western Blot

Western Blots were performed as described in our standard operating procedure. ^30^ HAP1 WT and *TIA1* KO were collected in RIPA buffer (25mM Tris-HCl pH 7.6, 150mM NaCl, 1% NP-40, 1% sodium deoxycholate, 0.1% SDS) (Thermo Fisher Scientific, cat. number 89901) supplemented with 1x protease inhibitor cocktail (MilliporeSigma, cat. number 78429). Lysates were sonicated briefly and incubated for 30 min on ice. Lysates were spun at ~110,000 x g for 15 min at 4°C and equal protein aliquots of the supernatants were analyzed by SDS-PAGE and Western Blot. BLUelf prestained protein ladder from GeneDireX (cat. number PM008-0500) was used.

Western Blots were performed with precast midi 4-20% Tris-Glycine polyacrylamide gels from Thermo Fisher Scientific (cat. number WXP42012BOX) ran with Tris/Glycine/SDS buffer from Bio-Rad (cat. number 1610772), loaded in Laemmli loading sample buffer from Thermo Fisher Scientific (cat. number AAJ61337AD) and transferred onto nitrocellulose membranes. Proteins on the blots were visualized with Ponceau S staining (Thermo Fisher Scientific, cat. number BP103-10) which is scanned to show together with individual Western Blot. Blots were blocked with 5% milk for 1 hr, and antibodies were incubated overnight at 4°C with 5% bovine serum albumin (BSA) (Wisent, cat. number 800-095) in TBS with 0,1% Tween 20 (TBST) (Cell Signalling Technology, cat. number 9997). Following three washes with TBST, the peroxidase conjugated secondary antibody was incubated at a dilution of ~0.2 μg/mL in TBST with 5% milk for 1 hr at room temperature followed by three washes with TBST. Membranes were incubated with Pierce ECL from Thermo Fisher Scientific (cat. number 32106) prior to detection with the HyBlot CL autoradiography films from Denville (cat. number 1159T41).

### Antibody screening by immunoprecipitation

Immunoprecipitation was performed as described in our standard operating procedure. ^31^ Antibody-bead conjugates were prepared by adding 1 μg of antibody to 500 μL of Pierce IP Lysis Buffer from Thermo Fisher Scientific (cat. number 87788) in a 1.5 mL microcentrifuge tube, together with 30 μL of Dynabeads protein A- (for rabbit antibodies) or protein G- (for mouse antibodies) from Thermo Fisher Scientific (cat. number 10002D and 10004D, respectively). Tubes were rocked for ~1 hr at 4°C followed by two washes to remove unbound antibodies.

HAP1 WT were collected in Pierce IP buffer (25 mM Tris-HCl pH 7.4, 150 mM NaCl, 1 mM EDTA, 1% NP-40 and 5% glycerol) supplemented with 1x protease inhibitor (Millipore Sigma, cat. number P8340). Lysates were rocked 30 min at 4°C and spun at 110,000 x g for 15 min at 4°C. 0.5 mL aliquots at 2.0 mg/mL of lysate were incubated with an antibody-bead conjugate for ~1 hr at 4°C. The antibody-bead conjugate can elute the protein of interest if the tested antibodies can successfully bind their antigen. The unbound fractions were collected and removed while the beads were subsequently washed three times with 1.0 mL of or IP lysis buffer and processed for SDS-PAGE and Western Blot on a precast midi 4-20% Tris-Glycine polyacrylamide gels. Prot-A: HRP (MilliporeSigma, cat. number P8651) was used as a secondary detection system at a dilution of 0.3 μg/mL for experiments where rabbit antibodies are used for both immunoprecipitation and its corresponding Western Blot.

### Antibody screening by immunofluorescence

Immunofluorescence was performed as described in our standard operating procedure. ^17^^–^^24^ HAP1 WT and *TIA1* KO were labelled with a CellTracker ^TM^ green (Thermo Fisher Scientific, cat. number C2925) or CellTracker ^TM^ deep red (Thermo Fisher Scientific, cat. number C34565) fluorescence dye, respectively. WT and KO cells were plated in 96-well plate with optically clear flat-bottom (Perkin Elmer, cat. number 6055300) as a mosaic and incubated for 24 hrs in a cell culture incubator at 37 ^o^C, 5% CO _2_. Cells were fixed in 4% paraformaldehyde (PFA) (Beantown chemical, cat. number 140770-10ml) in phosphate buffered saline (PBS) (Wisent, cat. number 311-010-CL) for 15 min at room temperature and then washed 3 times with PBS. Cells were permeabilized in PBS with 0.1% Triton X-100 (Thermo Fisher Scientific, cat. number BP151-500) for 10 min at room temperature and blocked with PBS containing 5% BSA, 5% goat serum (Gibco, cat. number 16210-064) and 0.01% Triton X-100 for 30 min at room temperature. Cells were incubated with IF buffer (PBS, 5% BSA, 0,01% Triton X-100) containing the primary TIA1 antibodies overnight at 4°C. Cells were then washed 3 × 10 min with IF buffer and incubated with corresponding Alexa Fluor 555-conjugated secondary antibodies in IF buffer at a dilution of 1.0 μg/mL for 1 hr at room temperature with DAPI (Thermo Fisher Scientific, cat. Number D3571) fluorescent stain, used to label the nuclei. Cells were washed 3 × 10 min with IF buffer and once with PBS.

Images were acquired on an ImageXpress micro widefield high-content microscopy system (Molecular Devices), using a 20x/0.45 NA air objective lens and scientific CMOS camera (16-bit, 1.97mm field of view), equipped with 395, 475, 555 and 635 nm solid state LED lights (Lumencor Aura III light engine) and bandpass emission filters (432/36 nm, 520/35 nm, 600/37 nm and 692/40 nm) to excite and capture fluorescence emission for DAPI, CellTracker ^TM^ green, Alexa fluor 555 and CellTracker ^TM^ deep red, respectively. Images had pixel sizes of 0.68 x 0.68 microns. Exposure time was set with maximal (relevant) pixel intensity ~80% of dynamic range and verified on multiple wells before acquisition. Since the IF staining varied depending on the primary antibody used, the exposure time was set using the most intensely stained well as reference. Frequently, the focal plane varied slightly within a single field of view. To remedy this issue, a stack of three images per channel was acquired at a z-interval of 4 microns per field and best focus projections were generated during the acquisition (MetaExpress v6.7.1, Molecular Devices). Segmentation was carried out on the projections of CellTracker ^TM^ channels using CellPose v1.0 on green (WT) and far-red (KO) channels, using as parameters the ‘cyto’ model to detect whole cells, and using an estimated diameter tested for each cell type, between 15 and 20 microns. ^32^ Figures were assembled with Adobe Photoshop (version 24.1.2) to adjust contrast then assembled with Adobe Illustrator (version 27.3.1).

## Data Availability

Zenodo: Antibody Characterization Report for TIA1, https://doi.org/10.5281/zenodo.7671718. ^26^ Zenodo: Dataset for the TIA1 antibody screening study, https://doi.org/10.5281/zenodo.7796012. ^27^
