# Supplementary material for: A guide to selecting high-performing antibodies for RNA-binding protein TIA1 for use in Western Blot, immunoprecipitation and immunofluorescence
Source: F1000Res. 2024 Apr 8;12:745. Originally published 2023 Jun 26. [Version 2] doi: 10.12688/f1000research.133645.2 (PMC11024596; doi:10.12688/f1000research.133645.2)
Supplement: Peer review history

# Reviewer report: Reviewer response for version 2

**DOI:** 10.5256/f1000research.164350.r264019

**Published:** 2024-04-17

**Authors:** Jozsef Gal

**Affiliations:** 1 Radiation Medicine, University of Kentucky, Lexington, Kentucky, USA; 2 Markey Cancer Center, University of Kentucky, Lexington, Kentucky, USA

I have no further comments to make.

Are sufficient details of methods and materials provided to allow replication by others?

Partly

Is the rationale for creating the dataset(s) clearly described?

Yes

Are the datasets clearly presented in a useable and accessible format?

Partly

Are the protocols appropriate and is the work technically sound?

Partly

Reviewer Expertise:

Biochemistry, cell biology, neurodegeneration

I confirm that I have read this submission and believe that I have an appropriate level of expertise to confirm that it is of an acceptable scientific standard.

# Reviewer report: Reviewer response for version 1

**DOI:** 10.5256/f1000research.146653.r204755

**Published:** 2024-03-20

**Authors:** Jozsef Gal

**Affiliations:** 1 Radiation Medicine, University of Kentucky, Lexington, Kentucky, USA; 2 Markey Cancer Center, University of Kentucky, Lexington, Kentucky, USA

This article by Fotouhi *et al.* describes the validation of 12 commercially available anti-TIA1 antibodies in immunoblotting, immunoprecipitation and immunofluorescence studies. The manuscript is well written. This is a very useful contribution to the field. It will certainly help researchers choose anti-TIA1 antibodies for future work. I only have minor issues and comments.

 General issues:

- The HAP1 cell line is of human origin. A lot of research employs murine models and cell lines. It would be very useful if the Authors could perform immunoblotting of a lysate from a murine cell line. I would recommend N2A/Neuro-2A cells. In my opinion, the antibodies that performed the best in HAP1 immunoblotting (ab140595, ab263945, NBP2-67203, 86050) would be sufficient.
- The Authors present their findings in a rather raw format without much interpretation. It would be great if the Authors would summarize their findings after each technique. It would also be very useful to provide a summary paragraph about which antibodies the Authors consider overall the best.

Minor comments:

- The term “Western blot” is kind of colloquial. I recommend the term “immunoblotting”, but I would like to leave it up to the Authors.
- TIA-1 has a close homolog in human cells, TIAR. To me, one of the most valuable contributions of this work is the ability to see which tested antibodies may cross-react with TIAR. Based on the immunoblotting image, A6237, GTX33545, and MA5-26474 may cross-react with TIAR. I think that it would be useful if the Authors would touch on this issue.
- In the Introduction: “... disrupting the function of TIA1 can lead to various diseases including cancer, autoimmune diseases and neurodegenerative disorders” – I recommend including further references in this paragraph for cancer and autoimmune diseases.
- Could you, please, check the molecular weight marker bands in Figure 1, A6237? The detected bands are a little lower than on several other immunoblotting images.
- It would be useful to discuss the extra bands (ARP40981, NBP3-13791) and the unexpected band sizes (NBP2-53336) in immunoblotting.
- In “Methods”, “Cell culture”: I believe that “2 mM L-glutamate (Wisent cat. number 609065)” is L-glutamine.
- “Methods”, “Antibody screening by Western Blot”: “... protease inhibitor cocktail mix (MilliporeSigma, cat. number 78429)”: I guess that you refer to the Halt™ Protease Inhibitor Cocktail, but that is a Thermo Fisher Scientific product.
- You wrote that the lysates were cleared with 110,000 x g centrifugation. Could you, please, confirm that? That is an ultracentrifugation speed. Lysates are usually cleared with centrifugal forces about an order of magnitude lower for lysate preparation for immunoblotting and immunoprecipitation.
- I advise against using the wavy line “~” in a method paper. I believe that it reads “about” or “approximately”. Instead, please just state the numbers.
- “Methods”, “Antibody screening by immunoprecipitation”: please state the dilution of the P8340 protease inhibitor cocktail.
- “Methods”, “Antibody screening by immunoprecipitation”: please describe how you eluted the proteins from the immunoprecipitation beads.
- “Methods”, “Antibody screening by immunofluorescence”: the DAPI staining is mentioned early in the protocol, and then later again, when the secondary antibody staining is described (without stating the DAPI concentration). I would not mention DAPI before the plating step because it could confuse the readers that it was used alongside the CellTracker stains.
- “DAPI (Thermo Fisher Scientific, cat. Number D3571)”: that, however, is a Sigma-Aldrich product.
- None of the TIA1 KO cells were completely dark in the immunofluorescence studies in Figure 3. It would be a good idea to point out that more stringent immunofluorescence conditions may be needed.

Are sufficient details of methods and materials provided to allow replication by others?

Partly

Is the rationale for creating the dataset(s) clearly described?

Yes

Are the datasets clearly presented in a useable and accessible format?

Partly

Are the protocols appropriate and is the work technically sound?

Partly

Reviewer Expertise:

Biochemistry, cell biology, neurodegeneration

I confirm that I have read this submission and believe that I have an appropriate level of expertise to confirm that it is of an acceptable scientific standard, however I have significant reservations, as outlined above.

# Response

**Published:** 2024-03-27

**Authors:** Kathleen Southern

**Affiliations:** McGill University, Canada

Thank you to Jozsef Gal for your extensive review of this article. We have submitted a modified version of this manuscript, with the requested modifications the authors found appropriate. We trust that the refinements made meet your expectations, enhance comprehensibility and address any concerns you might have had. Please see our responses to your specific comments below. 

 To address your general concerns, we’d first like to clarify that this study for TIA1 is part of a much larger collaborative initiative, seeking to characterize antibodies for all human proteins to address the antibody liability crisis. It would be very difficult to optimize every working parameter when in the process of trying to characterize antibodies for 20,000 proteins in the human proteome. That is why we have focused our initiative on testing antibodies in human cancer cell lines rather than murine models, but are well aware that many researchers employ these models. YCharOS presents the antibody characterization data to the scientific community, using a standardized protocol that allows researchers to select high-performing antibodies. This enables researchers who specialize in the target of interest to conduct further studies, including employing these high-performing antibodies on murine cell lines.

 Furthermore, the objective of this article, as well as the YCharOS initiative as a whole, is not to interpret the results nor summarize the performance of the antibodies tested. Given that it is formatted as a Data Note, it does not require the results to be discussed or concluded. WE’d like to re-iterate that YCharOS is a public-private organization whose mission is to characterize antibodies for every human protein and deliver the research as a collective good for the scientific community. That being said, we understand how this intention may be misinterpreted. A new version has been submitted, to include modifications to the title, and results&discussion section that ensure our goals are aligned and defined to all readers. Moreover, as the antibodies are tested under one specific set of conditions, summarizing the performance of the antibodies would be valid only under the precise experimental setup and cell line used.

 To address your minor comments, the authors have decided to stick with using the term Western blot to remain consistent with all other characterization reports. As for your concern regarding TIAR cross-reactivity, each commercial antibody tested was advertised on their respective catalogs as targeting TIA1, rather than TIAR. From what we’ve found in literature, TIAR is encoded by a different gene, being *TIAL *(1-3). For us to make accurate conclusions regarding the cross reactivity of the commercial antibodies, we would have had to prepare two KO cell lines; *TIA1* and *TIAL*.  

 In the newly submitted version of this article, additional references have been included to address TIA1’s role in various diseased states.

 The molecular weight marker has been confirmed the authors. Unlike other antibodies tested, A6237 recognizes bands in the WT cell line that do not disappear in the KO cell lines, indicating that it might not be properly targeting TIA1. When analyzing an antibody by Western blot, if it recognizes the target protein but produces extra bands as well, it is considered non-selective but specific. If it fails to recognize the target protein and produces unexpected bands, it is considered non-specific.  Guidelines on how to analyze antibody characterization data can be found in an editorial by Biddle et al., featured on the YCharOS gateway (4). In the newly submitted version of this article, we will include this reference.

 The following responses address your concerns regarding the methods section:

1. L-glutamate has been changed to L-glutamine.
2. The MilliporeSigma protease inhibitor cocktail mentioned is the one used in our standardized protocol. In our efforts to be more accurate, we have removed the word “mix” in the manuscript to reflect how it is written in the catalog.
3. WB: We found that ultra-centrifugation speed is required to pellet insoluble contaminants found in the lysates that would adhere to the bead-antibody conjugate, interfering with the detection of bound protein in Western blot. We’ve observed that Table-top centrifugation speeds do not adequately remove insoluble particles.
4. IP: the concentration for the protease inhibitor cocktail mix as well as a statement describing how the protein of interest can be eluded using the antibody-bead conjugation protocol has been added.
5. IF: we’ve relocated the mention of DAPI fluorescent stain to where secondary antibody conjugation is discussed to prevent confusion among readers. In this case, the DAPI stain was purchased from Thermo Fisher Scientific, which is why it is mentioned in the manuscript.

1. Tian Q, Streuli M, Saito H, Schlossman SF, Anderson P. A polyadenylate binding protein localized to the granules of cytolytic lymphocytes induces DNA fragmentation in target cells. Cell. 1991 Nov 1;67(3):629-39. doi: 10.1016/0092-8674(91)90536-8.
2. Velasco BR, Izquierdo JM. T-Cell Intracellular Antigen 1-Like Protein in Physiology and Pathology. Int J Mol Sci. 2022 Jul 16;23(14):7836. doi: 10.3390/ijms23147836.
3. Beck AR, Medley QG, O'Brien S, Anderson P, Streuli M. Structure, tissue distribution and genomic organization of the murine RRM-type RNA binding proteins TIA-1 and TIAR. Nucleic Acids Res. 1996 Oct 1;24(19):3829-35. doi: 10.1093/nar/24.19.3829.
4. Biddle MS and Virk HS. YCharOS open antibody characterisation data: Lessons learned and progress made [version 1; peer review: not peer reviewed]. *F1000Research* 2023, 12:1344 ( https://doi.org/10.12688/f1000research.141719.1)

# Reviewer report: Reviewer response for version 1

**DOI:** 10.5256/f1000research.146653.r195326

**Published:** 2023-09-07

**Authors:** Claudia Fallini

**Affiliations:** 1 George and Anne Ryan Institute for Neuroscience, University of Rhode Island, Kingston, Rhode Island, USA

In this manuscript, Fotouhi and colleagues characterized a panel of 12 antibodies for their ability to detect the RNA-binding protein TIA-1 in western blot, immunoprecipitation, and immunofluorescence assays. They selected the human cancer line HAP1 as model system as it produces high levels of the TIA-1 protein, and compared data to the isogenic TIA-1 KO line. The experimental procedures are well described and thorough, and the data are solidly presented. A couple of points that the authors could address are listed below:

 1. All experiments are performed on a human cell line. The authors should specify this limitation in their results and discussion section. Alternatively the authors could test the best performing antibodies on murine cells as well, as this would broaden the applicability of the study. 

 2. A summary table ranking the antibodies tested based on their performance on the three different assays would be helpful.

Are sufficient details of methods and materials provided to allow replication by others?

Yes

Is the rationale for creating the dataset(s) clearly described?

Yes

Are the datasets clearly presented in a useable and accessible format?

Partly

Are the protocols appropriate and is the work technically sound?

Yes

Reviewer Expertise:

Cell biology, neurodegenerative research

I confirm that I have read this submission and believe that I have an appropriate level of expertise to confirm that it is of an acceptable scientific standard.
